# Characterization of Three Heat Shock Protein Genes in *Pieris melete* and Their Expression Patterns in Response to Temperature Stress and Pupal Diapause

**DOI:** 10.3390/insects13050430

**Published:** 2022-05-05

**Authors:** Jing Zhang, Falak Naz Miano, Ting Jiang, Yingchuan Peng, Wanna Zhang, Haijun Xiao

**Affiliations:** 1 Institute of Entomology, Jiangxi Agricultural University, Nanchang 330045, China; jingz3801@jxau.edu.cn (J.Z.); anynazfalak47@gmail.com (F.N.M.); jiangting0315@126.com (T.J.); ycpeng@jxau.edu.cn (Y.P.); 2 School of Grassland Science, Beijing Forestry University, Beijing 100083, China

**Keywords:** *Pieris melete*, heat shock protein 70, summer and winter diapause, heat tolerance, cold tolerance

## Abstract

**Simple Summary:**

*Pieris melete*, a major pest of crucifers, undergoes obligatory diapause as pupae to survive unfavorable temperature extremes during hot summers and cold winters. Heat shock proteins 70 (Hsp70) participate in this process; however, little is known about the underlying changes in *Hsp70* expression both during the summer and winter diapause. The study aimed to investigate expression patterns of *Hsp70s* (*PmHsc70*/*PmHsp70a*, *b*) in response to diapause and short-term temperature stresses. The results showed that the expression of *PmHsc70* and *PmHsp70*b were upregulated both in summer and winter diapause. Heat shock significantly induced up-regulation of the three genes in both summer and winter diapause. In non-diapause pupae, none of the genes responded to cold or heat stress. Further, it was found that 39 °C for 30 min was the most sensitive heat stress condition for *PmHsc70* expressions in summer diapause and all three genes’ expressions in winter diapause. During summer diapause, the expression of the genes was up-regulated in response to high temperature acclimation at 31 °C. Meanwhile, only *PmHsp70a* and *PmHsp70b* were up-regulated when acclimated to a low temperature of 4 °C in winter diapause. In conclusion, the current results indicate that *PmHsp70s* plays a crucial role during both summer and winter diapause, in response to temperature stresses; and our findings may contribute to the increasing knowledge on seasonal diapause adaption.

**Abstract:**

Heat shock protein 70 genes participate in obligatory pupal diapause in *Pieris melete* to survive unfavorable conditions. In this study, three full-length cDNAs of *PmHsc70*, *PmHsp70a* and *PmHsp70b* were identified, and their expression patterns in response to diapause and short-term temperature stresses were investigated. Summer and winter diapause were induced in the pupae and non-diapause individuals were used as a control. The pupae from each diapause group were subjected to either hot or cold conditions and the expression levels of the HSP genes were measured. Our results showed that up-regulation of *PmHsc70* and *PmHsp70b* were detected both in summer and winter diapause, but not for *PmHsp70a*. Under cold stress, *PmHsp70a* and *PmHsp70b* were upregulated in summer and winter diapause, while heat shock significantly induced upregulation of all three genes. In non-diapause pupae, none of the genes responded to cold or heat stress. Furthermore, we found that incubation at 39 °C for 30 min was the most sensitive heat stress condition for *PmHsc70* expression in summer diapause. On the other hand, the same temperature was effective for *PmHsc70*, *PmHsp70a*, and *PmHsp70b* expression in winter diapause. During summer diapause, expression of all three genes was upregulated in response to high-temperature acclimation at 31 °C, but only *PmHsp70a* and *PmHsp70*b were upregulated when acclimated to a low temperature of 4 °C in winter diapause. These results suggest that the *PmHsc70*, *PmHsp70a*, and *PmHsp70b* respond differently to pupal diapause and temperature stress, and that *PmHsc70* is more sensitive to heat shock than to cold stress.

## 1. Introduction

Due to their ectothermic nature, insects are prone to be affected by many biotic and abiotic factors. Environmental temperature variation is one of the greatest challenges for insects, and adaptation to thermal stress is crucial for them to complete their life cycles [1]. The heredity mechanism involved in thermal tolerance in insects is the synthetic regulation of heat shock proteins (HSPs) [2]. HSPs, which were first discovered through their response to heat stress, are abundant in insects, and play a crucial role in enhancing environmental abiotic and biotic stress adaptation, as well as regulating development and dormancy [3]. Since the first HSP was discovered, their huge number and variations have been reported [4]. Based on their identified amino acid sequence, molecular mass, and functions, HSPs are classified into five major categories: Hsp100, Hsp90, Hsp70, Hsp60, and small (20–40 kD) HSPs [5].

Among the different categories of HSPs, the Hsp70s family is the most conserved and widely distributed among all organisms and has been studied in the context of environmental stress [6]. The Hsp70s family is divided into two sub-groups. The first group, the inducible Hsp70s, is induced rapidly in response to environmental stress and returns to a normal level in the absence of stress. The other group is the heat shock cognate protein 70 (Hsc70), which does not directly respond to environmental thermal stress but is constitutively expressed to maintain protein folding under normal conditions [7]. Hsp70s are essential molecules as they are involved in many important biological processes in various organisms, and their expression is markedly induced by all kinds of stresses, such as extremely high and low temperatures, insecticides, starvation, and heavy metals. For example, the transcription expression level of Hsp70s was significantly upregulated during exposure to thermal stress in *Monochamus alternatus* and *Aphelinus asychis* [8,9]. The expression of *Hsp70* was strongly upregulated by extremely high and low temperatures in *Drosophila melanogaster*, *Cotesia chilonis*, and *Liriomyza trifolii* [10,11,12]. Additionally, upregulation of *Hsp70* has been found in *Spodoptera litura* after exposure to heavy metals [13]. The role of *Hsp70* in resistance of insects against long periods of starvation was also investigated in *Rhodnius prolixus*, where it was found that when *RpHsp70* was knocked down by RNA interference in insects, the resistance against prolonged starvation decreased compared to the controls [14]. Furthermore, *HaHsc70* reportedly regulated larval development in *Helicoverpa armigera* through the ecdysone (20-hydroxyecdysone) signaling pathway [15].

Diapause is the stage of developmental arrest in insects that allows them to avoid harsh environments [16]. As one of the most important survival strategies, diapause allows insects to survive in unfavorable conditions. It is triggered by changes in daylight and temperature and is regulated by endogenous hormones and other molecular factors [4]. The mechanisms of diapause are complex; therefore, understanding the basic mechanism involved in diapause initiation and termination at a molecular level is helpful. Numerous studies have demonstrated that the regulation of HSPs is related to the types of diapause, with the most extensive research reported on winter diapause (WD) [4,17]. Due to the scarcity of reports on *Hsp* expression in response to summer diapause (SD), there is a gap in knowledge when it comes to the association between *Hsp* function and insect diapause. Research on *Drosophila triauraria* showed that the level of *DtHsp70* was similar between non-diapausing and diapausing insects [18], while expression of *Hsp70A* was upregulated in non-diapausing copepods compared to diapausing copepods [19]. Additionally, upregulation of *Hsp70* and *Hsp90* was found in diapausing individuals in *Chilo suppressalis* [20]. In *Sarcophaga crassipalpis*, *Hsp70* transcription was undetectable in non-diapausing individuals but was upregulated at pre-diapause stage. Furthermore, the expression of *Hsp90* was downregulated during diapause and remained at relatively low levels throughout the stage [21]. These studies suggest that different classes of HSPs may play different roles during diapause in different species.

The *Pieris melete* Ménétriés (Lepidoptera: Pieridae) experiences a multivoltine life cycle and is considered to be a serious pest for the cruciferous mountain plants in Jiangxi Province in China. Since this butterfly undergoes an SD and WD as a pupa, it may be an ideal model to study the bio-physiology and molecular mechanisms of diapause. The systematic investigation of the biology and physiology of diapause have been studied in more detail in *P. melete* both under laboratory and natural field conditions [22,23,24]; however, little is known about the molecular mechanistic regulation of diapause in this butterfly. Previous studies have shown that *PmHsp90* could be upregulated by heat- and cold- stress both in summer and winter arrest diapause [25]. Furthermore, differential gene expression profiles among non-diapause, SD, and WD showed that *PmHsp90* was induced in the species that experience diapause [26]. However, the characteristics of *Hsp70* genes in *P. melete* and their mRNA expression profiles related to diapause remain unknown.

In this study, the role of *PmHsc70*, *PmHsp70a*, and *PmHsp70b* in the SD and WD stages of *P.melete* was investigated. The full-length cDNA sequences of the three genes were cloned and the phylogenetic evolutionary relationship and sequence conservation characteristics were revealed. The expression patterns of the three *Hsp70s* genes were examined during SD and WD. Additionally, we systematically monitored the expression levels of *Hsp70* transcription in diapause individuals that were exposed to either extremely high or low-temperature treatments over different periods. Our results may help improve understanding of adaptation mechanism of *P. melete* during diapause in response to temperature stress, which in turn may benefit pest control and management of *P. melete*.

## 2. Materials and Methods

### 2.1. Insects

Five larvae of the *P. melete* were collected during the instar stage from cruciferous plants in the Northern area of Nanchang in China. Wooden hand-made net cages (30 × 30 × 35 cm) were used to transfer collected larvae for pupation and adult emergence. The adults were captured and frozen immediately in liquid nitrogen, and then stored at −80 °C for later use. The cloned samples of *PmHsc70*, *PmHsp70a*, and *PmHsp70b* were developed by reverse-transcription polymerase chain reaction (RT-PCR) from an RNA sample derived from *P. melete* adults.

### 2.2. RNA Isolation, cDNA Synthesis, and RACE Amplification

The total RNA was extracted using a TRIzol reagent (Invitrogen, Carlsbad, CA, USA), according to the standard procedures. The quality of the RNA sample was measured using 1% agarose gel electrophoresis and then tested with a NanoVue spectrophotometer (GE Healthcare, Wiesbaden, Germany). DNase I (Promega, Madison, WI, USA) was used to prevent DNA contamination. By using an M-MLV Reverse Transcriptase (Promega, Madison, WI, USA) reagent kit, the purified RNA (2 μg) was then used to synthesize cDNA, according to the manufacturer’s instructions. By using the conserved primers of the *β-actin* gene listed in Table S1, the cDNA quality was evaluated. Based on a local BLAST search of the transcriptomic database, three putative gene fragments were retrieved and verified in the NCBI database. For the 5′ and 3′-end of the three putative *Hsp70s* fragments, rapid amplification of cDNA ends (RACE) was performed to amplify the full-length cDNA sequence. The gene-specific primers enumerated in Table S1 were all designed by Primer 5.0 software (Premier Biosoft International, San Francisco, CA, USA). The 5′ first-strand cDNA templates of *P. melete* were amplificated using the SMART™ RACE Kit (Clontech, Mountain View, CA, USA). The RACE-PCR programs were conducted according to the manufacturer’s instructions. The purified DNA fragment was cloned into the pEASY-T cloning vector (TransGen, Beijing, China) and transformed into *Escherichia coli* DH5α (TransGen, Beijing, China) for sequencing. The positive clones were then selected and sequenced by BGI Tech. The sequences in this study have been submitted to the GenBank of the NCBI database (accession numbers MZ712581, MZ712582, and MZ712583 for *PmHsc70*, *PmHsp70*a, and *PmHsp70b*, respectively).

### 2.3. Sequence and Phylogenetic Analysis

The amino acid sequence alignment was done using DNAMAN software (6.0 version). The Pfam database for protein families (https://pfam.xfam.org/accessed on 19 November 2021) and the conserved domain database (http://blast.ncbi.nlm.nih.gov/Blast.cgi accessed on 17 Mar 2022) were used to predict conserved domains. The amino acid sequence of HSP70s from Lepidoptera [23], Diptera [5], Hymenoptera [4], and Coleoptera [4] were selected to estimate phylogeny by neighbor-joining method. Using MEGA (version X) (https://www.megasoftware.net/), the phylogenetic trees were set up using 1000 bootstrap replicates.

### 2.4. Preparation of Non-Diapause, Summer, and Winter Diapause Pupae

To induce diapause, *P.melete* were bred in the laboratory, and larvae were reared at 20 °C. A photoperiod of 14 L: 10 D was used to trigger the SD pupae; a photoperiod of 10 L: 14 D was used to induce the WD pupae, and an intermediate photoperiod of 12.5 L: 11.5 D was used for the non-diapause (ND) group. The ND, SD, and WD pupae were identified through the symptoms exhibited over six days of development (initiation stage of non-diapause or diapause) according to our previous study [22,25].

### 2.5. Cryogenic and Heat Shock Treatment on P. melete Pupae

Pupae in different forms of ND, SD, and WD were collected and categorized into four subgroups for cryogenic and heat treatment. The first subgroup underwent cryogenic chilling at 0 °C between 24–96 h in an incubator while the control pupae were kept at 18 °C. The second subgroup underwent a heat treatment between 31–43 °C for 30 min. The third subgroup underwent a heat treatment at 39 °C between 30 min to 4 h. The fourth subgroup included incubation of SD and WD pupae for a long period. To match the temperatures of the local environment, we set the temperature to 31 °C for 30 days for the SD pupae, and 18 °C for 60 days for the control treatment. Lastly, the WD pupae were incubated at a cool temperature of 4 °C for 90 days. For the fourth subgroup, the pre-diapause stage was defined as day 0, as diapause can only be identified 6 days after pupation. Samples for the diapause stage were therefore sampled 30–60 days after treatments (36–66 days after pupation). The post diapause stage sampling occurred 90 days after incubation (96 days after pupation). SD pupae were incubated at 18 °C and 31 °C, and WD pupae were incubated at 18 °C and 4 °C, with maintenance temperature until termination of diapause. SD and WD pupae were sampled at day 0, 30, 60, and 90 [24,25]. All pupae samples were frozen immediately in liquid nitrogen, and stored at −80 °C for later use.

### 2.6. Quantitative Real-Time PCR (qRT-PCR)

The relative mRNA expression levels of *PmHsc70*, *PmHsp70a*, and *PmHsp70b* in SD, WD, and ND groups in *P. melete* were analyzed using real-time fluorescence quantification PCR (qRT-PCR). The procedure included high-quality RNA extraction using TRIzol reagent (Invitrogen, Carlsbad, CA, USA), cDNA synthesis using the SuperScript III Reverse Transcriptase reagent test kit (Invitrogen, Carlsbad, CA, USA), and an oligo(dT) primer. The gene-specific primers for qRT-PCR were designed by Primer 3 (Table S1). *β-actin* (KU527138) and 18S ribosomal RNA (KU527139) were used as internal normalized genes. The qRT-PCR was performed using ABI Prism 7500 Fast System (Applied Biosystems, Foster, CA, USA) and reagent test kit from TIANGEN (SYBR Green SuperReal PreMix Plus, Beijing, China). Amplifications were carried out under the following conditions, initial denaturation at 95 °C for 10 min followed by 40 cycles of 5 s at 95 °C and for 30 s at 60 °C, followed by a melting curve stage (60–95 °C) to confirm gene-specific amplification. The amount of cDNA was determined using the 2^−ΔΔCT^ quantification method. The experiment was conducted with three biological treatments and three technical replicates.

### 2.7. Statistical Analysis

STATA software (Version 9.0; StataCorp., College Station, TX, USA) was used for data analysis. The average value of relative expression level compared to the internal normalized genes of *β-actin* and 18S RNA were analyzed using a one-way analysis of variance (ANOVA), and multiple comparisons by post hoc Bonferroni tests among different groups of cryogenic or heat treatments. All values are shown through mean ± standard error, with a significance criterion of *p* ≤ 0.05 used in all statistical comparisons.

## 3. Results

### 3.1. Sequence Analysis of PmHsc70, PmHsp70a, and PmHsp70b

**Figure 1:**
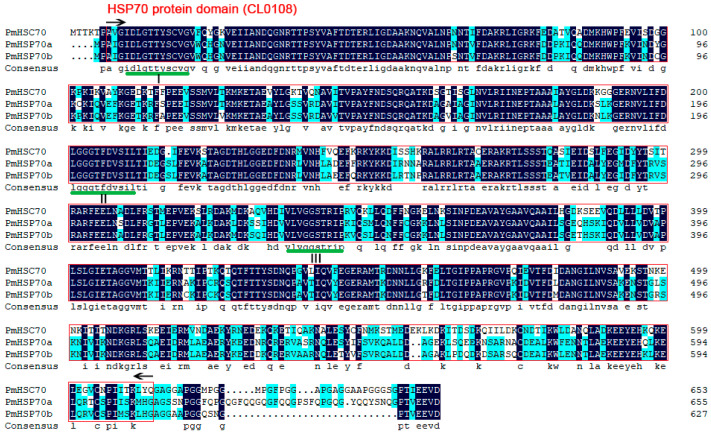
The alignment of amino acid sequences of PmHSC70, PmHSP70a, and PmHSP70b. The three signature sequences of HSP70 homologs are underlined. The conserved HSP70 protein domain (CL0108) is shown in the box. The final consensus sequence EEVD is at the C-terminus.

The full-length cDNA sequence of *PmHsc70*, *PmHsp70a*, and *PmHsp70b* were submitted to the NCBI GenBank database (accession numbers MZ712581, MZ712582, and MZ712583, respectively). The complete coding regions of *PmHsc70*, *PmHsp70a*, and *PmHsp70b* consisted of 1962, 1968, and 1884 bp open reading frames which encoded 654, 656, and 628 amino acids, with a deduced molecular weight of 71.6, 72.4, and 69.0 kDa, respectively (Figures S1–S3). Amino acid sequence analysis of PmHSC70, PmHSP70a, and PmHSP70b indicated that three signature sequences (underlined with I, II, III), the conserved HSP70 protein domain (CL0108), and the cytoplasmic characteristic motif EEVD were predicted (Figure 1).

### 3.2. Sequence Alignment and Phylogenetic Relationship Analysis

The amino acid sequences of PmHSC70, PmHSP70a, and PmHSP70b were aligned with the HSP70 proteins of other insect species. Homology analysis revealed that PmHSC70, PmHSP70a, and PmHSP70b were highly conserved in insects. PmHSC70 shared 99.67%, 96.56%, 96.23%, and 96.08% identity with *P. rapae* (XP_022130638.1), *Aricia agestis* (XP_041970467.1), *Vanessa tameamea* (XP_026489199.1), and *Ostrinia furnacalis* (XP_028170304.1), respectively (Figure S4). PmHSP70a shared 97.71%, 93.60%, 89.31%, and 88.55% identity with, *P. rapae* (QWV59544.1), *Zerene cesonia* (XP_038210277.1), *Spodoptera frugiperda* (KAG8115787.1), and *Melitaea cinxia* (XP_045456590.1), respectively (Figure S5). PmHSP70b exhibited 97.94%, 92.66%, 92.19%, and 91.72% identity with *P. rapae* (QWV59542.1), *M. cinxia* (XP_045456597.1), *M. cinxia* (XP_045456601.1), and *Pararge aegeria* (XP_039760955.1), respectively (Figure S6). The highly variable regions between PmHSP70 and other insect HSP70s with relatively low phylogeny were mostly located at the C-terminus.

**Figure 2:**
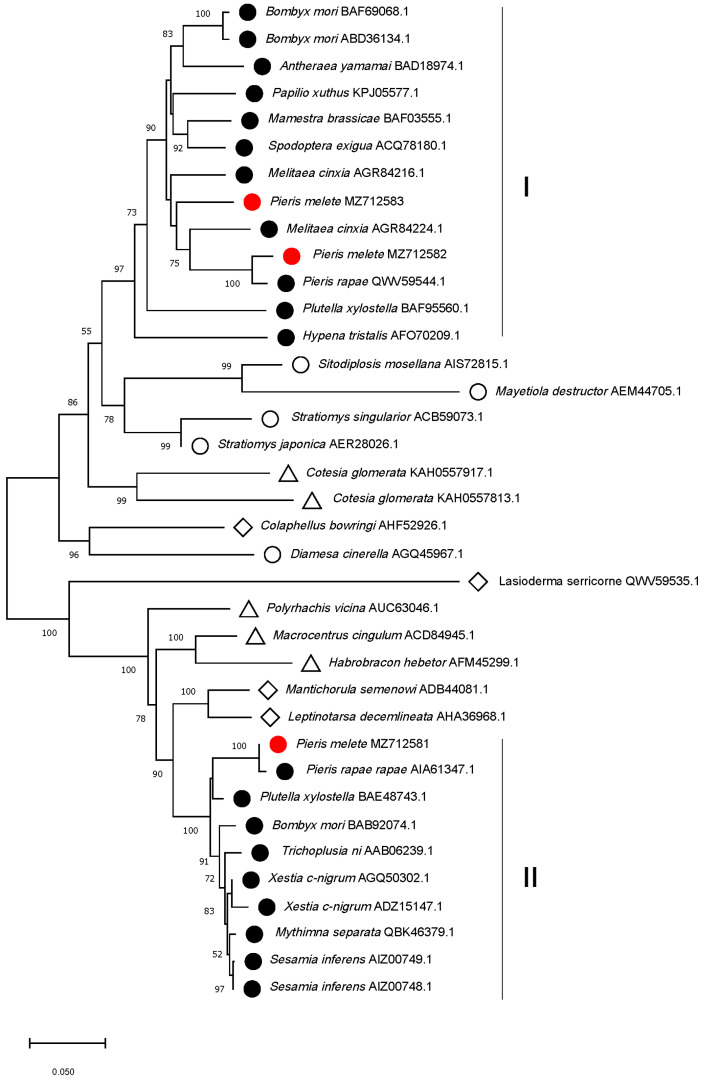
Phylogenetic neighbor-joining tree of PmHSC70, PmHSP70a, and PmHSP70b of *Pieris melete* and other insect HSPs using Mega X. The percentage bootstrap values obtained by 1000 repeated calculations were displayed at the nodes, and percentage values lower than 50% were collapsed. The PmHSC70, PmHSP70a, and PmHSP70b in our study were marked with a red solid circle. Lepidoptera, Coleoptera, Diptera, and Hymenoptera HSP70s from previous studies were labeled with a black solid circle, hollow rhombus, hollow circle, and hollow triangle, respectively.

Phylogenetic tree relationships showed that HSP70 proteins of the same order are almost clustered together. Moreover, Lepidopteran HSP70 proteins are classified into two main clusters (I and II). PmHSP70a and PmHSP70b belong to cluster I, and PmHSC70 belongs to cluster II (Figure 2). Annotation analysis of all HSP70 proteins of clusters I and II indicated that cluster I is mainly HSP70 proteins, while cluster II is mainly HSC70 proteins.

### 3.3. Expression Patterns of PmHsc70, PmHsp70a, and PmHsp70b in Pupae of ND, SD, and WD Pupae

**Figure 3:**
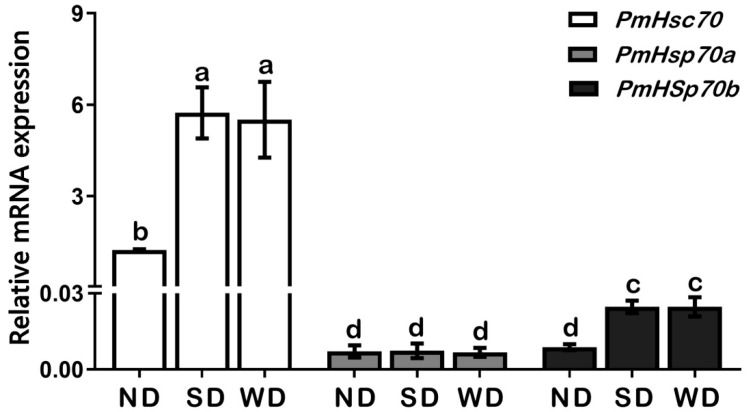
Relative mRNA levels of P*mHsc70*, *PmHsp70a*, and *PmHsp70b* in ND, SD, and WD pupae of *Pieris* *melete*. The relative expression levels (Mean ± SE) are normalized to *β-actin* and 18S rRNA. Different letters above the bar indicate significant differences by ANOVA and post hoc Bonferroni multiple comparison test (*p* < 0.05).

To clarify the role of *PmHsc70*, *PmHsp70a*, and *PmHsp70b* in pupal diapause, mRNA transcription levels were determined in ND, SD, and WD pupae. Our results showed that among the three genes, the expression level of *PmHsc70* is the highest, followed by *PmHsp70b*, and *PmHsp70a*. We found that compared to ND pupae, the expression levels of *PmHsc70* and *PmHsp70b* were upregulated significantly in both SD and WD groups; however, *PmHsp70a* did not display obvious differences in expression levels between these three groups (Figure 3).

### 3.4. Expression Patterns of PmHsc70, PmHsp70a, and PmHsp70b in ND, SD, and WD Pupae at 0 °C Cryogenic Chilling

**Figure 4:**
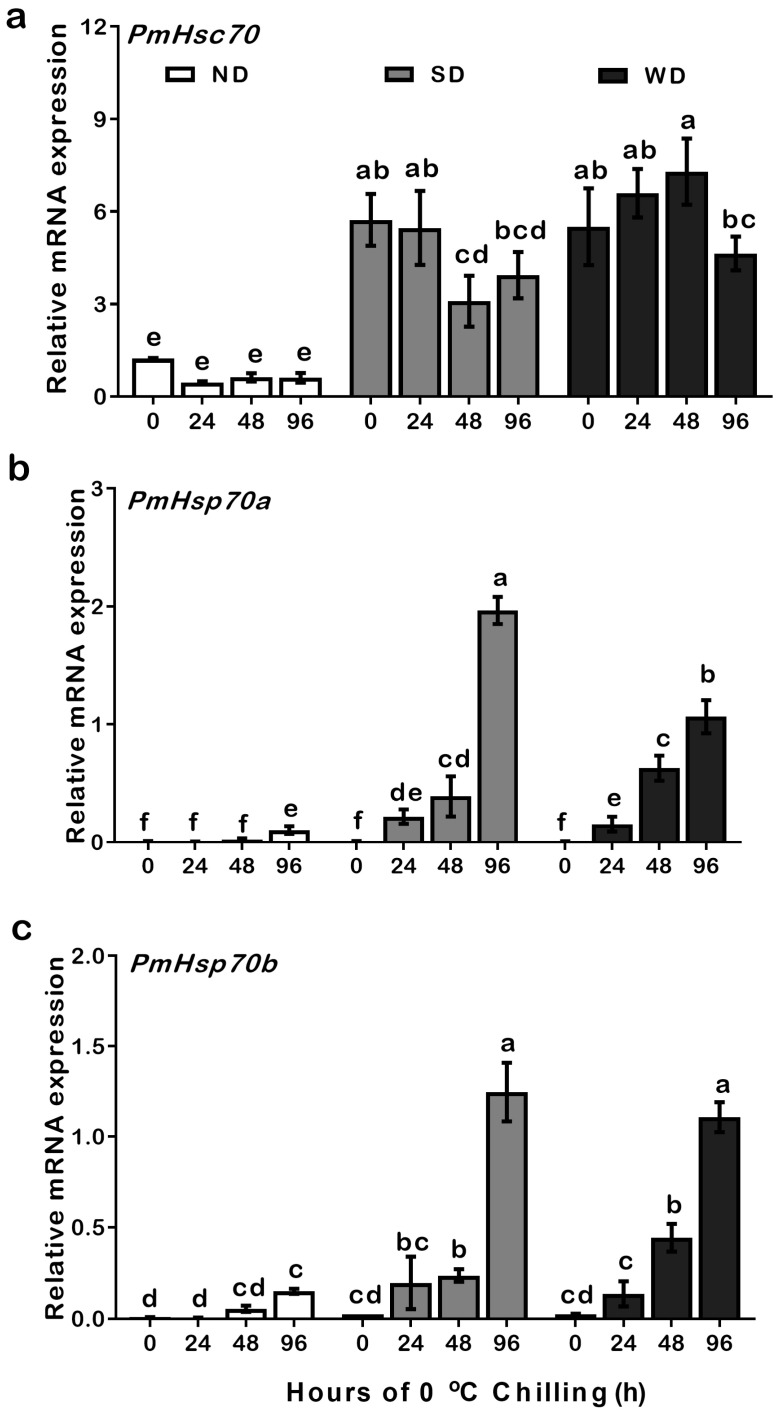
Relative mRNA expression levels of *PmHsc70* (**a**), *PmHsp70a* (**b**), and *PmHsp70b* (**c**) in response to low-temperature cryogenic chilling at 0 °C in ND, SD, and WD pupae of *Pieris* *melete*. The relative expression levels (Mean ± SE) are normalized to *β-actin* and 18S rRNA. Letters above the bar indicate significant differences by ANOVA and post hoc Bonferroni multiple comparison test (*p* < 0.05).

To test the effects of cold temperature on mRNA transcription levels in ND, SD, and WD pupae, we transferred the samples into incubators at 0 °C for a period between 24–96 h of cryogenic chilling. Our qRT-PCR results suggested that the expression level of *PmHsc70* was upregulated significantly in SD and WD compared to ND pupae (Table S2). In SD pupae, the expression level of *PmHsc70* did not change significantly after treatment for 24 h and 96 h but was significantly downregulated after 48 h. Cryogenic chilling of WD pupae showed an upward trend in *PmHsp70a* expression from 0 to 48 h, but decreased significantly after 96 h (Figure 4a).

The expression trends of *PmHsp70a* and *PmHsp70b* in response to low temperatures were similar. Both relative mRNA expression values of *PmHsp70a* and *PmHsp70b* were upregulated significantly in both SD and WD compared to ND pupae during the 24–96 h cryogenic treatment (Table S2). The expression of *PmHsp70a* and *PmHsp70b* did not change in ND pupae within 48 h of the cold treatment but increased significantly after 96 h. The expression of *PmHsp70a* and *PmHsp70b* in SD or WD pupae started to upregulate after 24 h, with a slight increase between 24 and 48 h, followed by major upregulation after 96 h (Figure 4b,c).

### 3.5. Expression Patterns of PmHsc70, PmHsp70a, and PmHsp70b in ND, SD and WD Pupae during Heat Treatments

**Figure 5:**
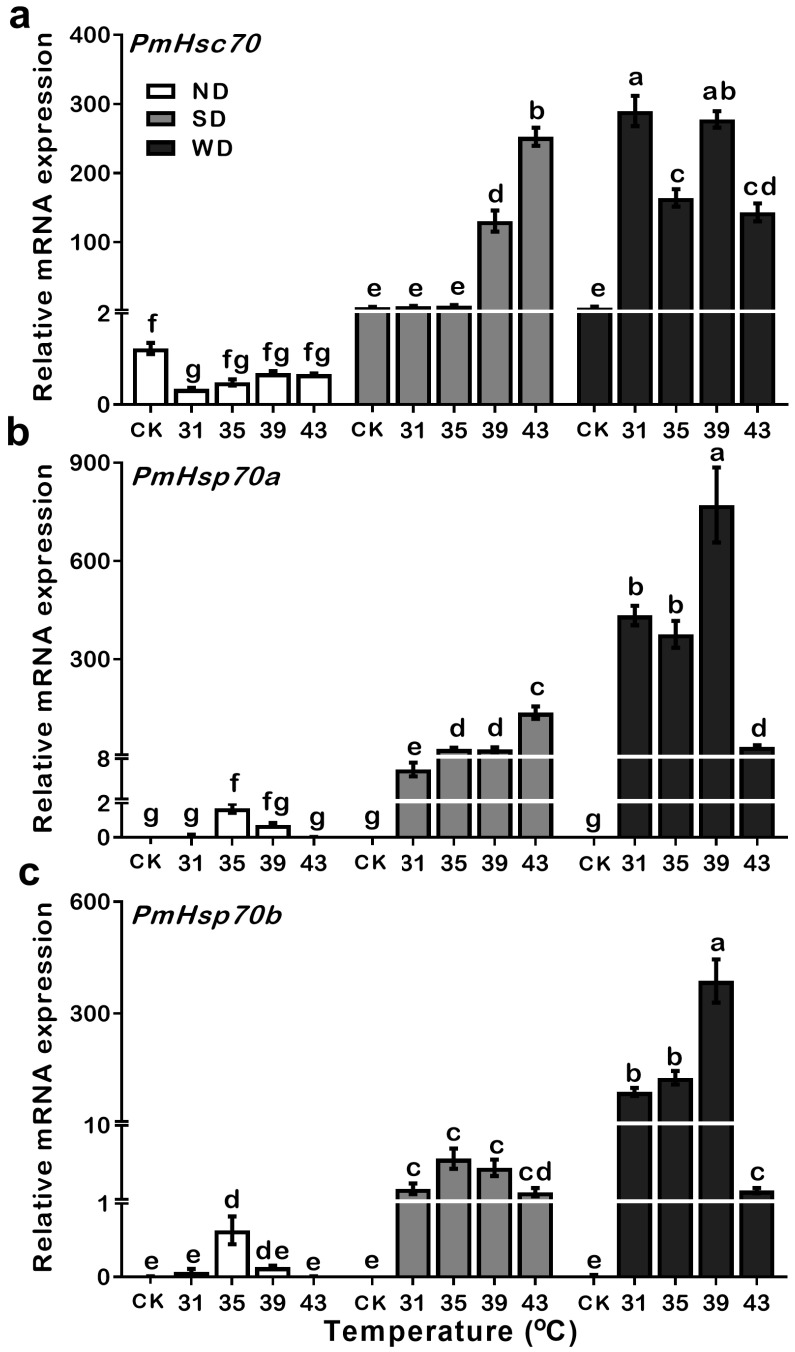
Relative mRNA expression levels of *PmHsc70* (**a**), *PmHsp70a* (**b**), and *PmHsp70b* (**c**) in response to heat treatment in ND, SD, and WD pupae of *Pieris melete*. The relative expression levels (Mean ± SE) are normalized to *β-actin* and 18S rRNA. Different letters above the bar indicate significant differences by ANOVA and hoc Bonferroni multiple comparison test (*p* < 0.05).

To examine whether the genes of *PmHsc70*, *PmHsp70a*, and *PmHsp70b* are heat responsive, the pupae of ND, SD, and WD were incubated at temperatures from 31 to 43 °C for 30 min and their expression levels were measured. Compared to the control treatment (CK), the expression of *PmHsc70* was stable in ND pupae after heat treatment. However, in SD pupae, the expression levels of *PmHsc70* were upregulated multiple times between 39–43 °C over 30 min. Notably, in WD pupae, the expression of *PmHsc70* was increased significantly regardless of the temperature (Table S3). These results indicate that *PmHsc70* was sensitive to heat shock stress in both summer and winter diapause, especially in WD pupae (Figure 5a).

Expression levels of *PmHsp70a* in the ND pupae were significantly upregulated after exposure to 35 °C for 30 min, but not at other temperature treatments. Expression levels of the *PmHsp70a* in SD pupae were significantly upregulated at all temperatures. Similar results were also found in the WD pupae; however, with multiple changes in the expression level of *PmHsp70a*, a dramatic decrease in gene expression occurs following temperature shift to 43 °C (Figure 5b, Table S3). The same mode of *PmHsp70b* expression level can also be observed in ND, SD, and WD pupae (Figure 5c, Table S3), suggesting that the upregulation of *PmHsp70a* and *PmHsp70b* are easily triggered by short bursts of heat in response to diapause.

### 3.6. Expression Patterns of PmHsc70, PmHsp70a, and PmHsp70b in ND, SD, and WD Pupae at 39 °C

**Figure 6:**
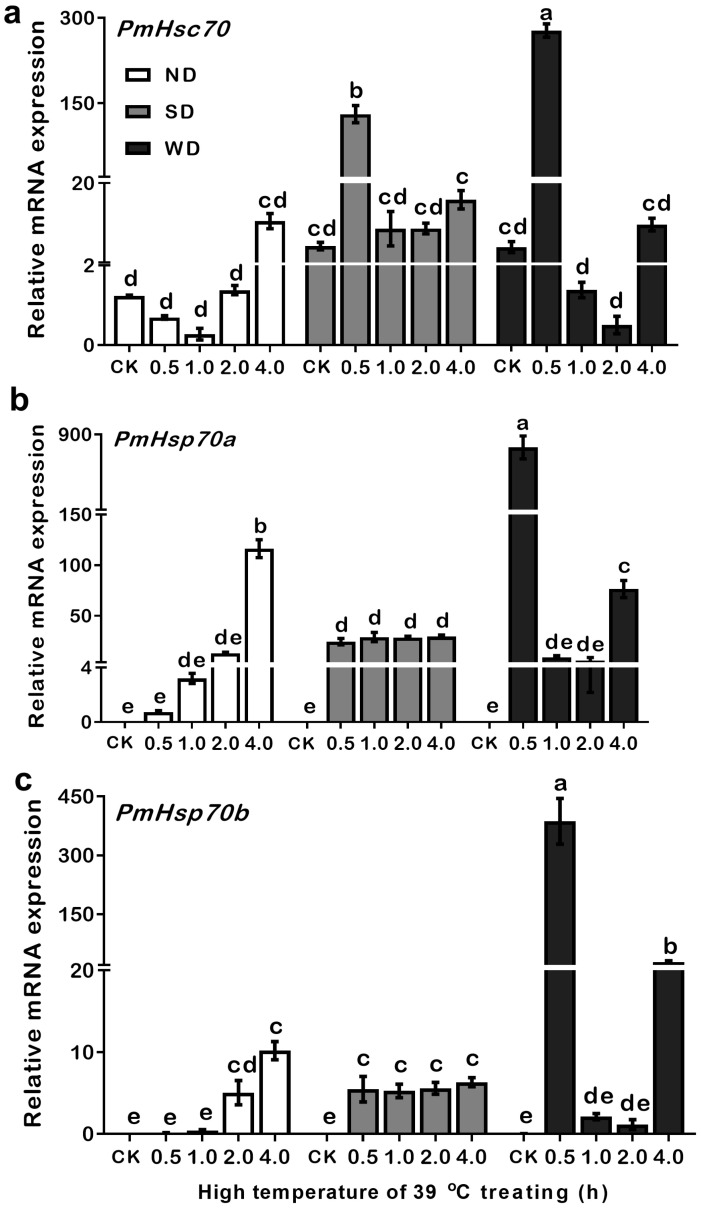
Relative mRNA expression levels of *PmHsc70* (**a**), *PmHsp70a* (**b**), and *PmHsp70b* (**c**) in response to 39 °C of heat stress in non-diapause (ND), summer diapause (SD), and winter diapause (WD) pupae of *Pieris* *melete*. The relative expression levels (Mean ± SE) are normalized to *β-actin* and *18S rRNA*. Different letters above the bar indicate significantly differences by ANOVA and post hoc Bonferroni multiple-comparison (*p* < 0.05).

The fact that in WD pupae, the highest expression levels of *PmHsc70*, *PmHsp70a*, and *PmHsp70b* were induced under 39 °C, suggests that 39 °C may be the most sensitive temperature condition for regulating these three genes. Therefore, the expressions levels of each gene were detected under 39 °C at different time points (0.5–4.0 h) (Figure 6).

Results indicated that in the ND pupae, compared with the CK, the expression level of *PmHsc70* decreased after a 30 min and 1 h treatment, but increased gradually between 2 to 4 h. However, the expression levels of *PmHsp70a* and *PmHsp70b* both increased with time in ND pupae, and their expression was significantly increased 16,387 and 1167 times over 4 h, respectively. In SD pupae, compared to the control, *PmHsc70* was significantly upregulated at 39 °C for a 30 min heat treatment, while *PmHsp70a* and *PmHsp70b* were all significantly upregulated at 39 °C among different time points. The most notable feature in WD pupae is that the expression levels of *PmHsc70*, *PmHsp70a*, and *PmHsp70b* increased when incubated at 39 °C for 30 min, and the expression levels were decreased when the incubation time was prolonged to 1 or 2 h with a recovery period after 4 h (Figure 6, Table S4).

### 3.7. Expression Patterns of PmHsc70, PmHsp70a, and PmHsp70b during Procedures of Summer and Winter Diapause Development

**Figure 7:**
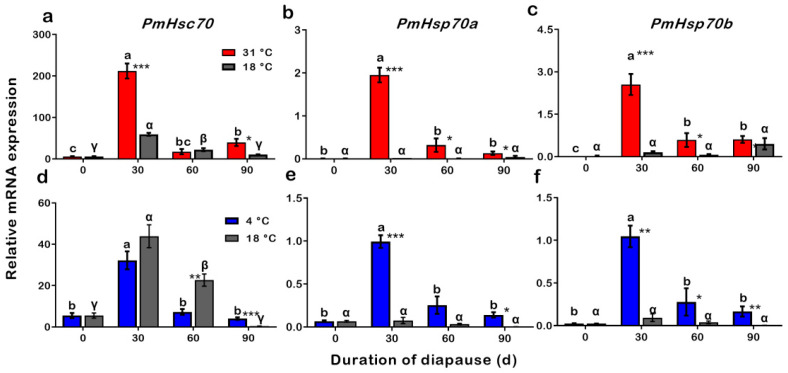
Relative expression analysis of *PmHsc70*, *PmHsp70a*, and *PmHsp70b* mRNA transcription levels in SD (**a**–**c**) and WD (**d**–**f**) pupae that were incubated at different temperatures during diapause development. The relative expression levels (Mean ± SE) are normalized to *β-actin* and 18S rRNA. Different letters or Greek alphabet (α, β, γ) above the bar indicate significant differences by ANOVA and post hoc Bonferroni multiple comparison test (*p* < 0.05) during diapause development at each temperature. The significant differences between 31 °C (SD), 4 °C (WD), and 18 °C (control) treatments are represented with an asterisk *. * *p* < 0.05, ** *p* < 0.01, and *** *p* < 0.001.

To test expression levels of *PmHsc70*, *PmHsp70a*, and *PmHsp70b* during development of SD and WD (0, 30, 60, 90 d), SD pupae were incubated at 18 °C and 31 °C, and WD pupae were incubated at 18 °C and 4 °C. The qRT-PCR results revealed that during diapause programming, the mRNA expression levels of *PmHsc70*, *PmHsp70a*, and *PmHsp70b* dramatically changed in both SD and WD. The mRNA expression of *PmHsc70*, *PmHsp70a*, and *PmHsp70b* were all significantly upregulated when acclimated at 31 °C of SD development in SD pupae, compared to diapause pupae incubated at 18 °C. It is worth noting that the highest expression of *PmHsc70*, *PmHsp70a*, and *PmHsp70b* in SD pupae was after 30 days of diapause and after being transferred to 31 °C (Figure 7a–c). On the other hand, only *PmHsp70a* and *PmHsp70*b were upregulated when acclimated to a low temperature of 4 °C in WD (Figure 7d–f).

## 4. Discussion

The ability to adapt to fluctuations in temperature is critical for insect survival, and the main defense strategy that ectothermic animals use for thermotolerance is the overly abundant HSPs [4]. In this study, the members of *Hsp70s* family (*PmHsc70*, *PmHsp70a*, and *PmHsp70b*) were identified and cloned from *P. melete*. The amino acid sequences in PmHSC70, PmHSP70a, and PmHSP70b are highly conserved and contain the typical HSP70 motifs (Figure 1). Phylogenetic analysis showed that these three HSPs were divided into two categories and that the HSP70s of *P. melete* are highly homologous to those in other Lepidoptera insects (Figure 2), suggesting a long history of independent evolution.

### 4.1. Expressions of PmHsc70, PmHsp70a, and PmHsp70b Related to Diapause and Cold Stress

In this study, the expression levels of both *PmHsc70* and *PmHsp70b* were significantly upregulated in SD and WD pupae than that in ND pupae (Figure 3). Cryogenic chilling at 0 °C for 48 h triggered *PmHsc70* downregulation in SD pupae while promoting the upregulation of *PmHsp70a* and *PmHsp70b* in SD and WD pupae in comparison to ND individuals (Figure 4). As one of the most common proteins in eukaryotic cells, HSPs are crucial for survival and adaptation to harsh thermal and cryogenic stresses [27], and the up- or down-regulation of HSP genes is crucial for insect survival in harsh environments during diapause [4,10,28]. For instance, the expression of *CsHsc70* slightly decreased during cryogenic treatment irrespective of the state of diapause in *C. suppressalis* [20]. In diapausing *Leptinotarsa decemlineata*, *LdHsp70A* was not significantly induced during the diapause stage, and the expression of *LdHsp70B* was undetectable [29]. In *D. melanogaster*, *Hsc70-1* was not induced by cold, whereas *Hsp70Aa* was [30]. Using RNAi to suppress *Hsp23* and *Hsp70* expression in *S. crassipalpis* did not change the result on whether diapause was triggered or how long it lasted; however, it did influence pupal survival at low temperatures [28]. *HSP70* expression in the onion maggot *Delia antiqua* was exceptionally low in ND pupae, however, it was upregulated following cryogenic and heat treatments [31]. Cryogenic treatment at 0–4 °C and subsequent recovery, upregulated *Hsp70* expression, but not *Hsc70* in the corn earworm *Helicoverpa zea* [2]. All these studies demonstrate that *Hsc70* and *Hsp70* show a different response to cold stress. In addition, 24–48 h in cryogenic treatment at 0 °C resulted in a slight upregulation of *PmHsp70a* and *PmHsp70b* in SD and WD pupae, which increased after 96 h. This may suggest that diapause pupae have a stronger tolerance to cold stress than ND pupae (Figure 4). Furthermore, the stable expression of *PmHsc70* under all conditions suggests that *Hsc70* is not sensitive to cryogenic treatment.

### 4.2. Expressions of PmHsc70, PmHsp70a, and PmHsp70b Related to Heat Shock

Heat shock upregulation of *HSP70* under high temperatures is well documented in many organisms; however, different expression patterns under various stages of stress are exhibited in different insects. In the diamondback moth, for example, the expression of *PxHsc70* was high for both chlorpyrifos-susceptible and chlorpyrifos-resistant strains when larvae were grown at 25 °C and showed a relatively stable response to heat treatment. However, expression of *PxHsp70s* in response to heat treatment was upregulated in chlorpyrifos-susceptible strains compared to chlorpyrifos-resistant strains [32]. Expression estimated by northern blotting and RT-PCR showed that heat treatment at 45 °C for 3 h did not affect elevating *Hsp70* mRNA expression in adipose or neural tissues of *Locusta migratoria* [33]. Translation and transcription expression of *Alhsc70* in *Apolygus lucorum* was significantly upregulated at 40 °C [34]. In *Bombyx mori*, upregulation of *BmHsp70-1*, *BmHsp70-2*, and *BmHsp70-3* occurred in a heat treatment between 37–42 °C [35]. In addition, high temperatures upregulated the expression level of *Hsp70* in other organisms, such as *Drosophila buzzatii* [36], *Chrysomela aeneicollis* [37], *Nilaparvata lugens* [38], *Frankliniella occidentali*s [39], and *Bemisia tabaci* [40]. All these examples illustrate that *Hsp70s* may play a critical role in the adaptation to high-temperature stress. Similarly, in our study, *PmHsc70*, *PmHsp70a*, and *PmHsp70b* transcription levels were significantly upregulated in heat treatments both in SD and WD pupae (Figure 5). This may suggest that upregulation of *Hsp70s* expression levels is a critical adaptation mechanism to extreme thermal stress. In addition, during SD of *P. melete*, the upregulation of *PmHsc70* was only affected following heat treatment at 39 °C and 43 °C, and the expression level of *PmHsp70a* and *PmHsp70b* showed an increasing trend between 31–43 °C. In WD pupae, our results indicated that the expression levels of *PmHsc70*, *PmHsp70a*, and *PmHsp70b* reached their peak at 39 °C within 30 min of heat treatment, followed by a sharp decrease at 43 °C (Figure 5). This may suggest that SD pupae have obtained stronger tolerance to extreme thermal stress than WD and ND pupae.

Upregulation of HSPs may be critical for seasonal adaptation in insects; however, *Hsp* expression cannot be sustained during heat and cold stressors. Although *Hsp70* upregulation increases the thermotolerance of insects, there are also costs, such as impacted growth, fecundity, and other biological parameters [41]. One study showed that in *Pteromalus puparu*, a significant upregulation of *Pphsc70* expression was caused by heat treatment; however, the continued exposure to thermal stress and a return to normal temperature caused a gradual downregulation to even lower levels of expression than those of control pupae [42]. Furthermore, results in a study on the mite *Neoseiulus barkeri* revealed that the expression of two *NbHsp70s* was significantly elevated at 42 °C but then sharply decreased after 4 h of heat treatment [43]. In this study, our results revealed that *Hsp70s* upregulation at 39 °C of heat treatment reached peak levels within 30 min in diapause pupae, but was downregulated when the incubation time was prolonged to 1–2 h with recovery after 4 h in WD pupae (Figure 6).

### 4.3. Expressions of PmHsc70, PmHsp70a, and PmHsp70b during Summer and Winter Diapause

The differential expression of *HSP*s was strongly affected by environmental stimulates and varied significantly during diapause, which suggests the importance of seasonal adaptation to thermal stress for survival during that time [4,17]. In this investigation of *P. melete*, the expression of all three HSPs was upregulated in SD pupae incubated at 31 °C. The expression of *PmHsp70a* and *PmHsp70b* was also upregulation in WD pupae incubated at 4 °C compared to the control (Figure 7). In terms of the expression dynamics of *Schsc70*, they remained unaltered during different diapause stages. Upregulation of *Schsp70* in *S. crassipalpis* started during the pre-diapause stage and remained high throughout the entire process of diapause. During the deep diapause stage, however, heat treatment did not further impact the expression level of *Schsp70*. Moreover, within 12 h after diapause was terminated, the expression of *Schsp70* was suspended [28]. An embryo-specific form of *AlHsp70* was induced during embryonic development in *Austrofundulus limnaeus*, and it was upregulated during diapause stage II [44].

The functions of insect *Hsp*s are quite complex, with different HSPs playing distinct roles during diapause, even within the same species. For example, in *Sitodiplosis mosellana* diapause larvae, *Hsp70* may contribute to heat tolerance while *Hsp90* plays a key role in cold tolerance [45]. In a previous study of *P.* *melete*, cryogenic chilling and thermal stress instantly induced considerable upregulation of *PmHsp90*, both in SD and WD pupae; however, *PmHsp90* expression was relatively stable in ND pupae [25], similar to the expression level of *PmHsp70* in this study (Figure 4 and Figure 5). *Hsp*s are a large family of genes; therefore, whether other *HSPs* (e.g., small *HSPs*) show a similar trend in *P. melete* responding to temperature stress is still unknown, and further study on the role of *HSPs* can help us to better recognize the regulating mechanism of cryogenic or thermal stress adaptation in *P. melete*, and eventually improve the management of this pest.

## 5. Conclusions

In summary, this study isolated and characterized three genes, *PmHsc70*, *PmHsp70a*, and *PmHsp70b*, that are highly conserved in the *P. melete*. In ND pupae, none of the three genes responded to cold or heat stress. Under cold stress, *PmHsp70a* and *PmHsp70b* were up-regulated in SD and WD. Heat shock significantly induced upregulation of the three *PmHsp70s* in both SD and WD. At 39 °C for 30 min was the most sensitive heat stress condition for *PmHsc70* expressions in SD and the three gene expressions in WD. During SD, expression of three *PmHsp70s* was upregulated in response to high temperature acclimation at 31 °C. Meanwhile, only *PmHsp70a* and *PmHsp70b* were upregulated when acclimated to a low temperature of 4 °C in WD. Overall, the three genes investigated respond differently to pupal diapause and temperature stress, and that *PmHsc70* is more sensitive to heat than to cold stress. Our findings may contribute to a better understanding of diapause-related cryogenic and thermal stress adaptation.

## Data Availability

The full-length cDNAs sequence of *PmHsc70*, *PmHsp70a*, and *PmHsp70b* were submitted to the NCBI GenBank database (accession no. MZ712581, MZ712582, and MZ712583, respectively).

## Supplementary Materials

The following supporting information can be downloaded at: https://www.mdpi.com/article/10.3390/insects13050430/s1, Table S1: Primers used in RT-PCR, RACE or qRT-PCR, Table S2: ANOVA results for the differential relative mRNA levels of PmHsc70, PmHsp70a, and PmHsp70b in response to 0 °C cryogenic chilling for different duration in non-diapause (ND), summer diapause (SD), and winter diapause (WD) pupae of P. melete, Table S3: ANOVA results for the differential relative mRNA levels of PmHsc70, PmHsp70a, and PmHsp70b in response to different heat stress temperatures in non-diapause (ND), summer diapause (SD), and winter diapause (WD) pupae of P. melete, Table S4: ANOVA results for the differential relative mRNA levels of PmHsc70, PmHsp70a, and PmHsp70b in response to 39 °C heat stress at different time points in non-diapause (ND), summer diapause (SD), and winter diapause (WD) pupae of P. melete, Figure S1: Nucleotide and deduced amino acid sequences of PmHsc70, Figure S2: Nucleotide and deduced amino acid sequences of PmHsp70a, Figure S3: Nucleotide and deduced amino acid sequences of PmHsp70b, Figure S4: Sequence alignment of PmHSC70 with HSP70 proteins of Pieris rapae (XP_022130638.1), Aricia agestis (XP_041970467.1), Vanessa tameamea (XP_026489199.1) and Ostrinia furnacalis (XP_028170304.1), Figure S5: Sequence alignment of PmHSP70a with HSP70 proteins of Pieris rapae (QWV59544.1), Zerene cesonia (XP_038210277.1), Spodoptera frugiperda (KAG8115787.1), and Melitaea cinxia (XP_045456590.1), Figure S6: Sequence alignment of PmHSP70b with HSP70 proteins of Pieris rapae (QWV59542.1), Melitaea cinxia (XP_045456597.1), Melitaea cinxia (XP_045456601.1), and Pararge aegeria (XP_039760955.1).
