# Supplementary material for: Characterization of Three Heat Shock Protein Genes in Pieris melete and Their Expression Patterns in Response to Temperature Stress and Pupal Diapause
Source: Insects. 2022 May 5;13(5):430. doi: 10.3390/insects13050430 (PMC9146241; doi:10.3390/insects13050430)
Supplement: Supplementary file 1 [file insects-13-00430-s001.zip › insects-1687975-supplementary.pdf]

Supplementary Tables and Figures

Table S1. Primers used in RT-PCR, RACE or qRT-PCR.

Primer name	Primers nucleotide sequence (5' to 3')
Actin-F	CATCTACGAGGGTTACGC
Actin-R	CATCTGTTGGAAGGTGGA
PmHsc70 3'F1	TAACACGACCATCCCCACCAAGCA
PmHsc70 3'F2	CCCAGACTTTCACGACCTATTCCGACA
PmHsc70 5'R1	CGGGTTGGTTGTCGGAATAGGTCG
PmHsc70 5'R2	GCCTCGTCAGGGTTGATGGACTTGTT
PmHsc70 5'R3	ACCTCCCAAGTGAGTGTCTCCAGCAGT
PmHsp70a 3'F1	GCTGAGGCGTATTTGGGTAGTTCGGTC
PmHsp70a 3'F2	TCTACACCAGGGTTTCTCGGGCACG
PmHsp70a 5'R1	AGCGTCTATTTCAATGGTGGCTTCCGT
PmHsp70a 5'R2	CAGCGTCTCTGACCGAACTACCCAAAT
PmHsp70b 3'F1	CGCCGCTGTCACTTGGCATAGAAACTG
PmHsp70b 3'F2	GAATGCTGGCAGAGGCTGAACGATACA
PmHsp70b 5'R1	ATTCCAGCAATAACCCCAGCATCCTT
PmHsp70b 5'R2	TGAAATAGGCGGGCACTGTGACGAC
*PmHsp70*b 5'R3	GATGGTGTTGTTCGGTTGCCCTGGT
PmHsc70 ORF F	GTGATAGAAACAAACTATACGC
PmHsc70 ORF R	TAATTGAGAGGATTACATCGAG
PmHsp70a ORF F	AGCGAATACTAAACAAGTGG
PmHsp70a ORF R	AGTACATGATCATGGATTGC
PmHsp70b ORF F	CAGTGATATTGTTTATCAGAAG
PmHsp70b ORF R	CATATGTTTTAACACTGCAGTC
*PmActi*n F	CAGGGAAAAGATGACCCAGA
PmActin R	GCAGAGCGTAACCCTCGTAG
Pm18S F	AATGCCGCTTGAATATTTCG
Pm18S R	TTTCGCTGATGTTCGTCTTG
PmHsp70a F	TCCGCGAGGAGTACCTAAGA
PmHsp70a R	GGCTTCAGCCAACATACGAT
PmHsp70b F	CGCTGTCACTTGGCATAGAA
PmHsp70b R	TACAGCAGGCTGGTTGTCAG
PmHsc70 F	TGCTGGAGACACTCACTTGG
PmHsc70 R	TTCAATGCTAGCCTGTGTCG

Table S2. ANOVA results for the differential relative mRNA levels of *PmHsc70*, *PmHsp70a*, and *PmHsp70b* in response to 0°C cryogenic chilling for different duration in non-diapause (ND), summer diapause (SD), and winter diapause (WD) pupae of *P. melete*.

Gene	Source	df	MS	F	Sig.
	Type of Diapause/nondiapause	2	89.221	61.06	0.0000
PmHsc70	Duration of chilling	3	2.463	1.69	0.1967
	Type of diapause * Duration	6	3.364	2.30	0.0675
	Type of Diapause/nondiapause	2	1.179	173.31	0.0000
PmHsp70a	Duration of chilling	3	1.940	285.09	0.0000
	Type of diapause * Duration	6	0.581	85.320	0.0000
	Type of Diapause/nondiapause	2	0.554	51.72	0.0000
PmHsp70b	Duration of chilling	3	1.208	112.74	0.0000
	Type of diapause * Duration	6	0.219	20.42	0.0000

Table S3. ANOVA results for the differential relative mRNA levels of *PmHsc70*, *PmHsp70a*, and *PmHsp70b* in response to different heat stress temperatures in non-diapause (ND), summer diapause (SD), and winter diapause (WD) pupae of *P. melete*.

Gene	Source	df	MS	F	Sig.
	Type of Diapause/nondiapause	2	115701.732	427.04	0.0000
PmHsc70	Temperature	4	27773.736	102.51	0.0000
	Type of diapause * Temperature	8	24349.575	89.87	0.0000
	Type of Diapause/nondiapause	2	465025.497	147.22	0.0000
PmHsp70a	Temperature	4	91058.6437	28.83	0.0000
	Type of diapause * Temperature	8	110894.094	35.11	0.0000
	Type of Diapause/nondiapause	2	71344.563	100.69	0.0000
PmHsp70b	Temperature	4	25683.945	36.25	0.0000
	Type of diapause * Temperature	8	24889.397	35.13	0.0000

Table S4. ANOVA results for the differential relative mRNA levels of *PmHsc70*, *PmHsp70a*, and *PmHsp70b* in response to 39°C heat stress at different time procedures in non-diapause (ND), summer diapause (SD), and winter diapause (WD) pupae of *P. melete*.

Gene	Source	df	MS	F	Sig.
PmHsc70	Type of Diapause/nondiapause	2	11621.189	145.18	0.0000
	Hours at 39 °C	4	30762.762	384.32	0.0000
	Type of diapause * Hours at 39 °C	8	11545.736	144.24	0.0000
PmHsp70a	Type of Diapause/nondiapause	2	109542.901	41.39	0.0000
	Hours at 39 °C	4	110593.749	41.79	0.0000
	Type of diapause * Hours at 39 °C	8	118227.429	44.67	0.0000
PmHsp70b	Type of Diapause/nondiapause	2	31874.158	46.78	0.0000
	Hours at 39 °C	4	28642.747	42.04	0.0000
	Type of diapause * Hours at 39 °C	8	29046.4764	42.63	0.0000

```
1     TGATAATTCTTTTGAAGTGATAGAAACAAACTATACGCAATGACTACTAAAACACCAGCGGTAGGTATTGACTTGGGTACCACATACTCG

1                                                M  T  T  K  T  P  A  V  G  I  D  L  G  T  T  Y  S
91    TGCGTGGGCGTGTTCCAGTACGGTAAGGTGGAGATCATCGCCAACGATCAGGGCAACAGGACTACACCTTCATATGTTGCCTTCACAGAC
31     C  V  G  V  F  Q  Y  G  K  V  E  I  I  A  N  D  Q  G  N  R  T  T  P  S  Y  V  A  F  T  D
181   ACCGAACGTCTCATCGGAGATGCCGCCAAGAACCAGGTGGCCCTGAACCCAAACAACACCATCTTTGATGCCAAACGTCTCATCGGGCGT
61     T  E  R  L  I  G  D  A  A  K  N  Q  V  A  L  N  P  N  N  T  I  F  D  A  K  R  L  I  G  R
271   AAGTTCGAAGATGCCACAGTTCAGGCTGACATGAAACACTGGCCTTTTGAGGTAATCAGTGATGGTGGAAAACCAAAGATCAAGGTTGCA
91     K  F  E  D  A  T  V  Q  A  D  M  K  H  W  P  F  E  V  I  S  D  G  G  K  P  K  I  K  V  A
361   TACAAGGGTGAAGACAAAACATTCTTCCCAGAGGAAGTCAGTTCAATGGTATTAACAAAAATGAAAGAAACAGCTGAAGTTTACCTTGGC
121    Y  K  G  E  D  K  T  F  F  P  E  E  V  S  S  M  V  L  T  K  M  K  E  T  A  E  V  Y  L  G
451   AAAACGGTGCAGAATGCAGTAATTACAGTGCCAGCATACTTTAATGACTCCCAAAGACAGGCCACAAAAGATTCTGGTACAATCTCTGGT
151    K  T  V  Q  N  A  V  I  T  V  P  A  Y  F  N  D  S  Q  R  Q  A  T  K  D  S  G  T  I  S  G
541   CTGAACGTTCTCCGAATCATCAATGAACCAACTGCTGCTGCGATTGCATATGGTCTTGACAAGAAAGGAGGTGGAGAACGTAACGTCCTT
181    L  N  V  L  R  I  I  N  E  P  T  A  A  A  I  A  Y  G  L  D  K  K  G  G  G  E  R  N  V  L
631   ATTTTCGATCTTGGCGGTGGTACTTTTGATGTATCCATCCTGACCATCGAGGATGGTATCTTTGAAGTGAAGTCCACTGCTGGAGACACT
211    I  F  D  L  G  G  G  T  F  D  V  S  I  L  T  I  E  D  G  I  F  E  V  K  S  T  A  G  D  T
721   CACTTGGGAGGTGAGGACTTTGACAACCGCATGGTCAACCACTTTGTACAGGAGTTCAAGCGGAAGTACAAAAAGGACATTTCCTCCCAC
241    H  L  G  G  E  D  F  D  N  R  M  V  N  H  F  V  Q  E  F  K  R  K  Y  K  K  D  I  S  S  H
811   AAGAGGGCCCTGCGTAGGTTGAGGACAGCTTGTGAGCGCGCGAAGAGGACTCTTTCCTCGTCGACACAGGCTAGCATTGAAATCGACTCT
271    K  R  A  L  R  R  L  R  T  A  C  E  R  A  K  R  T  L  S  S  S  T  Q  A  S  I  E  I  D  S
901   CTGTTTGAGGGTATCGATTACTACACATCCATCACCCGGGCGCGTTTCGAAGAACTGAACGCCGACTTATTTAGATCTACCATGGAGCCT
301    L  F  E  G  I  D  Y  Y  T  S  I  T  R  A  R  F  E  E  L  N  A  D  L  F  R  S  T  M  E  P
991   GTAGAGAAGTCCCTCCGTGACGCCAAGATGGACAAGGCGCAAGTGCACGACATTGTCCTCGTCGGAGGTTCCACTCGTATCCCAAGAGTG
331    V  E  K  S  L  R  D  A  K  M  D  K  A  Q  V  H  D  I  V  L  V  G  G  S  T  R  I  P  R  V
1081  CAGAAGCTCCTTCAAGACTTCTTCAATGGCAAGGAGTTGAACAAGTCCATCAACCCTGACGAGGCCGTAGCCTACGGAGCGGCCGTTCAG
361    Q  K  L  L  Q  D  F  F  N  G  K  E  L  N  K  S  I  N  P  D  E  A  V  A  Y  G  A  A  V  Q
1171  GCCGCCATCTTGCACGGTGATAAGTCTGAAGAAGTACAGGATCTGCTCCTGTTGGATGTGACCCCGCTGTCCCTCGGTATCGAGACTGCA
391    A  A  I  L  H  G  D  K  S  E  E  V  Q  D  L  L  L  L  D  V  T  P  L  S  L  G  I  E  T  A
1261  GGCGGTGTCATGACCACCTTGATTAAGCGTAACACGACCATCCCCACCAAGCAAACCCAGACTTTCACGACCTATTCCGACAACCAACCC
421    G  G  V  M  T  T  L  I  K  R  N  T  T  I  P  T  K  Q  T  Q  T  F  T  T  Y  S  D  N  Q  P
1351  GGTGTGCTTATTCAAGTATTTGAGGGTGAGCGTGCCATGACCCGAGACAACAACCTTCTCGGTAAATTTGAGCTCACTGGTATTCCCCCA
451    G  V  L  I  Q  V  F  E  G  E  R  A  M  T  R  D  N  N  L  L  G  K  F  E  L  T  G  I  P  P
1441  GCGCCCCGTGGTGTCCCACAAATCGAAGTTACTTTCGACATTGACGCCAACGGTATCCTTAACGTATCGGCTGTCGAAAAATCTACCAAC
481    A  P  R  G  V  P  Q  I  E  V  T  F  D  I  D  A  N  G  I  L  N  V  S  A  V  E  K  S  T  N
1531  AAGGAGAACAAGATCACGATCACCAATGACAAGGGTCGTCTGTCCAAAGAAGAGATCGAGCGTATGGTCAACGACGCTGAGAAATACAGG
511    K  E  N  K  I  T  I  T  N  D  K  G  R  L  S  K  E  E  I  E  R  M  V  N  D  A  E  K  Y  R
1621  AACGAAGATGAGAAACAGAAGGAAACCATTCAGGCTAAGAACGCGCTCGAATCTTACTGCTTCAACATGAAGTCTACCATGGAGGATGAG
541    N  E  D  E  K  Q  K  E  T  I  Q  A  K  N  A  L  E  S  Y  C  F  N  M  K  S  T  M  E  D  E
1711  AAGCTTAAGGACAAAATCACAGACTCCGACAAGCAAATCATTTTGGACAAATGCAACGACACCATCAAATGGTTGGACGCTAACCAGCTG
571    K  L  K  D  K  I  T  D  S  D  K  Q  I  I  L  D  K  C  N  D  T  I  K  W  L  D  A  N  Q  L
1801  GCTGACAAGGAAGAATATGAGCACAAGCAGAAGGAACTGGAGGGTGTGTGCAACCCGATTATAACCAAGCTTTACCAGGGTGCAGGTGGA
601    A  D  K  E  E  Y  E  H  K  Q  K  E  L  E  G  V  C  N  P  I  I  T  K  L  Y  Q  G  A  G  G
1891  GCCCCCGGAGGCATGCCCGGTGGTATGCCAGGCTTCCCTGGCGGAGCGCCTGGAGCCGGTGGAGCAGCCCCTGGTGGCGGATCCGGACCC
631    A  P  G  G  M  P  G  G  M  P  G  F  P  G  G  A  P  G  A  G  G  A  A  P  G  G  G  S  G  P
1981  ACCATCGAAGAGGTTGATTAAACTATTTGAATTATACATTCCACTTTTCACATTGTAAGATCTTCTCGATGTAATCCTCTCAATTAATAA
661    T  I  E  E  V  D
2071  ATGAATGCAACTGAAAAAAAAAAAAAAAAAAAAAAAAAAAAAA
```

Figure S1. Nucleotide and deduced amino acid sequences of *PmHsc70*.

```
1     ATCAGTATTTGAAAAGACAAACAAGCGAACAGTAAAAGTGAAGTCGGTGTCAATACCAAGTTATACAAGCGAATACTAAACAAGTGGAGC
91    GAATACTAAACAAGTGGATCTATTTATTGAACGAGTGATAAAATAATTAAAAAAATCATCAAAATGCCAGCTATTGGAATTGATCTTGGA
1                                                                       M  P  A  I  G  I  D  L  G
181   ACCACATACTCATGTGTGGGAGTGTGGCAACATGGAAACGTGGAAATCATCGCAAACGACCAAGGCAACCGAACAACACCATCATACGTT
31     T  T  Y  S  C  V  G  V  W  Q  H  G  N  V  E  I  I  A  N  D  Q  G  N  R  T  T  P  S  Y  V
271   GCATTCACAGACACAGAGAGACTTATTGGAGATGCCGCTAAGAACCAGGTCGCTTTGAACCCGAATAACACAGTGTTCGACGCAAAGCGA
61     A  F  T  D  T  E  R  L  I  G  D  A  A  K  N  Q  V  A  L  N  P  N  N  T  V  F  D  A  K  R
361   CTGATCGGCAGAAAATTCGACGACCCCAAGATTCAGCAAGATATGAAACACTGGCCTTTCAAGGTCATCAACGACTACGGTAAATGTAAA
91     L  I  G  R  K  F  D  D  P  K  I  Q  Q  D  M  K  H  W  P  F  K  V  I  N  D  Y  G  K  C  K
451   ATTCAGGTGGAATTCAAGGGCGAAACAAAACGATTTTCTCCGGAAGAAATCAGCAGCATGGTGCTCACGAAGATGAAGGAAACTGCTGAG
121    I  Q  V  E  F  K  G  E  T  K  R  F  S  P  E  E  I  S  S  M  V  L  T  K  M  K  E  T  A  E
541   GCGTATTTGGGTAGTTCGGTCAGAGACGCTGTTATAACGGTACCAGCTTACTTCAACGACTCCCAACGCCAGGCAACCAAGGATGCGGGT
151    A  Y  L  G  S  S  V  R  D  A  V  I  T  V  P  A  Y  F  N  D  S  Q  R  Q  A  T  K  D  A  G
631   GCGATCGCCGGTATCAACGTCTTAAGGATAATAAATGAACCCACCGCTGCCGCTTTGGCATATGGCCTGGACAAAAGTCTCAAAGGAGAA
181    A  I  A  G  I  N  V  L  R  I  I  N  E  P  T  A  A  A  L  A  Y  G  L  D  K  S  L  K  G  E
721   CGTAACGTCTTGATTTTTGATCTCGGAGGCGGTACCTTTGATGTATCCATCCTAACTATCGACGAGGGATCATTGTTTGAAGTTAAAGCT
211    R  N  V  L  I  F  D  L  G  G  G  T  F  D  V  S  I  L  T  I  D  E  G  S  L  F  E  V  K  A
811   ACTGCCGGCGATACGCATCTCGGTGGTGAGGACTTTGACAACAGACTAGTCAACCACCTGGCTGATGAATTCTTCCGAAAGTACAAGAAG
241    T  A  G  D  T  H  L  G  G  E  D  F  D  N  R  L  V  N  H  L  A  D  E  F  F  R  K  Y  K  K
901   GACATAAGGAATAATGCTCGCGCTCTGCGTCGACTACGAACCGCTGCTGAAAGAGCTAAACGGACCTTATCATCGAGCACGGAAGCCACC
271    D  I  R  N  N  A  R  A  L  R  R  L  R  T  A  A  E  R  A  K  R  T  L  S  S  S  T  E  A  T
991   ATTGAAATAGACGCTCTCTACGAAGGAATGGACTTCTACACCAGGGTCTCTCGGGCACGGTTTGAGGAATTAAACTCGGATTTGTTCCGC
301    I  E  I  D  A  L  Y  E  G  M  D  F  Y  T  R  V  S  R  A  R  F  E  E  L  N  S  D  L  F  R
1081  GGTACTCTGGAACCAGTCGAGAAGGCCCTTAAGGATGCTAAGCTTGACAAGAGTTCTATTCACGATGTAGTGTTGGTTGGCGGTTCTACT
331    G  T  L  E  P  V  E  K  A  L  K  D  A  K  L  D  K  S  S  I  H  D  V  V  L  V  G  G  S  T
1171  CGTATTCCAAAAATTCAGAGCATGCTGCAAAACTTCTTCTGTGGTAAGAAATTAAACCTCTCCATCAACCCCGATGAAGCAGTTGCCTAT
361    R  I  P  K  I  Q  S  M  L  Q  N  F  F  C  G  K  K  L  N  L  S  I  N  P  D  E  A  V  A  Y
1261  GGCGCTGCGGTTCAAGCAGCTATCTTAAGTGGGGAACAGCACAGTAAAATCCAGGATGTACTGCTAGTAGACGTTGCTCCATTGTCATTG
391    G  A  A  V  Q  A  A  I  L  S  G  E  Q  H  S  K  I  Q  D  V  L  L  V  D  V  A  P  L  S  L
1351  GGTATTGAGACTGCAGGAGGAGTAATGACGAAGATCATTGAGCGGAATGCGAAGATTCCATGCAGACAGTCACAAACCTTCACCACATAC
421    G  I  E  T  A  G  G  V  M  T  K  I  I  E  R  N  A  K  I  P  C  R  Q  S  Q  T  F  T  T  Y
1441  TCGGACAACCAACCGGCCGTTACGATCCAGGTGTATGAGGGTGAACGAGCAATGACTAAAGACAACAACCTGCTCGGGCGATTTGATCTT
451    S  D  N  Q  P  A  V  T  I  Q  V  Y  E  G  E  R  A  M  T  K  D  N  N  L  L  G  R  F  D  L
1531  ACTGGAATACCACCAGCTCCGCGAGGAGTACCTAAGATAGACGTCACCTTCGATTTAGATGCCAATGGAATCTTAAACGTATCCGCAAAA
481    T  G  I  P  P  A  P  R  G  V  P  K  I  D  V  T  F  D  L  D  A  N  G  I  L  N  V  S  A  K
1621  GAAAACAGTACTGGCCTAAGCAAGAACATCGTGATCAAGAATGATAAGGGCCGTCTTTCGCAAGCTGAGATCGATCGTATGTTGGCTGAA
511    E  N  S  T  G  L  S  K  N  I  V  I  K  N  D  K  G  R  L  S  Q  A  E  I  D  R  M  L  A  E
1711  GCCGAAAGGTACAAAGAAGAAGACAACCGCCAAAGAGAAAGAGTAGCATCTCGCAATCAACTAGAATCCTACATCTTTAGTGTGAAACAG
541    A  E  R  Y  K  E  E  D  N  R  Q  R  E  R  V  A  S  R  N  Q  L  E  S  Y  I  F  S  V  K  Q
1801  GCTTTGGACGATGCTGGTGAAAAACTCAGTCAAGAAGAAAAGAACAGTGCTCGAAATGCGTGTGATGAAGCTCTCAAATGGTTCGAAAAC
571    A  L  D  D  A  G  E  K  L  S  Q  E  E  K  N  S  A  R  N  A  C  D  E  A  L  K  W  F  E  N
1891  AATACCTTAGCGGAAAAAGAAGAATATGAACACCAGCTTAAGGAATTACAAAGGACTTGCTCTCCAATAATTAGCAAGATGCACGGAGCT
601    N  T  L  A  E  K  E  E  Y  E  H  Q  L  K  E  L  Q  R  T  C  S  P  I  I  S  K  M  H  G  A
1981  GGTTCCAGTAACCCAGGTGGCTTCCAGCCCGGACAAGGATTCCAACAAGGGCAAGGATTCCAGCAAGGACCAAGTTTTCAACCAGGACAA
631    G  S  S  N  P  G  G  F  Q  P  G  Q  G  F  Q  Q  G  Q  G  F  Q  Q  G  P  S  F  Q  P  G  Q
2071  GGCTACCAACAATACAGCAACCAAGGTCCCACAGTAGAGGAAGTAGATTAAAATGACATAAAATCCAATTAAATCTAGTACAACTTTGTA
661    G  Y  Q  Q  Y  S  N  Q  G  P  T  V  E  E  V  D
2161  AATAGTATTGTTAAGTTTCTGACTGATTAGTTCTTAAATATTTATTATTAGCAATCCATGATCATGTACTGCAATCCATGATCATGTACT
2251  GCTTAATCTTAGAATAAATTAAGTAAAAAAAAAAAAAAAAAAAAAAAAAAAAAA
```

Figure S2. Nucleotide and deduced amino acid sequences of *PmHsp70a*.

```
1     AGACTAATTCGAAATACGAGAGAGTGAACAAGTGATATTCAGTGAGAATCGAGGCATATTATACTATCTTGCGAATATTTTCCTGTGAAG
91    TTTGTTGAAGTATTATATATTCAGTGATATTGTTTATCAGAAGACACAATGCCAGCTATTGGAATTGATCTTGGTACAACCTACTCTTGC
1                                                      M  P  A  I  G  I  D  L  G  T  T  Y  S  C
181   GTTGGTGTTTGGCAACATGGAAATGTGGAGATCATCGCAAATGACCAGGGCAACCGAACAACACCATCGTACGTCGCATTTACGGACACG
31     V  G  V  W  Q  H  G  N  V  E  I  I  A  N  D  Q  G  N  R  T  T  P  S  Y  V  A  F  T  D  T
271   GAGCGTCTGATCGGCGATGCTGCTAAGAACCAAGTAGCTTTAAACCCTAGCAACACAGTGTTCGATGCGAAAAGGCTGATCGGCCGTAAA
61     E  R  L  I  G  D  A  A  K  N  Q  V  A  L  N  P  S  N  T  V  F  D  A  K  R  L  I  G  R  K
361   TTCGACGATCCCAAAATTCAACAGGACATGAAACATTGGCCTTTTAAAGTTATCAATGACTGCGGTAAGCCTAAGATCCAGGTTGAATTC
91     F  D  D  P  K  I  Q  Q  D  M  K  H  W  P  F  K  V  I  N  D  C  G  K  P  K  I  Q  V  E  F
451   AAAGGCGAAACAAAACGATTTGCCCCAGAAGAAGTCAGCAGCATGGTTTTGGTGAAAATGAAGGAGACAGCTGAGGCCTACCTTGGATCA
121    K  G  E  T  K  R  F  A  P  E  E  V  S  S  M  V  L  V  K  M  K  E  T  A  E  A  Y  L  G  S
541   TCAGTGCGAGATGCCGTCGTCACAGTGCCCGCCTATTTCAATGATTCACAGCGTCAGGCGACCAAGGATGCTGGGGTTATTGCTGGAATA
151    S  V  R  D  A  V  V  T  V  P  A  Y  F  N  D  S  Q  R  Q  A  T  K  D  A  G  V  I  A  G  I
631   AATGTTTTACGTATCATAAACGAGCCCACTGCCGCTGCTCTGGCATATGGCCTAGACAAAAATCTAAAGGGCGAACGCAATGTCCTTATT
181    N  V  L  R  I  I  N  E  P  T  A  A  A  L  A  Y  G  L  D  K  N  L  K  G  E  R  N  V  L  I
721   TTCGATCTTGGTGGCGGCACATTTGACGTTTCCATTTTGACGATCGACGAGGGTTCGCTTTTTGAAGTGAAGGCCACGGCCGGAGACACA
211    F  D  L  G  G  G  T  F  D  V  S  I  L  T  I  D  E  G  S  L  F  E  V  K  A  T  A  G  D  T
811   CACCTTGGAGGTGAAGACTTTGACAACAGGCTCGTTAACCACCTAGCGGAGGAGTTCCAACGAAAATACAAGAAAGATTTGCGCACGAAC
241    H  L  G  G  E  D  F  D  N  R  L  V  N  H  L  A  E  E  F  Q  R  K  Y  K  K  D  L  R  T  N
901   CCACGCGCATTGCGACGCCTCCGCACCGCCGCAGAGCGGGCCAAGCGCACTTTATCATCTAGCACCGAGGCCACGGTTGAAATCGACGCG
271    P  R  A  L  R  R  L  R  T  A  A  E  R  A  K  R  T  L  S  S  S  T  E  A  T  V  E  I  D  A
991   CTATACGAAGGTATTGATTTCTACACTCGCGTATCTCGGGCTCGGTTCGAAGAATTAAACGCTGATCTATTCCGGGGCACCCTCGAACCA
301    L  Y  E  G  I  D  F  Y  T  R  V  S  R  A  R  F  E  E  L  N  A  D  L  F  R  G  T  L  E  P
1081  GTGGAAAAAGCATTGAAAGATGCTAAGATGGATAAGAGCCAAATACATGACGTGGTTCTCGTCGGAGGGTCCACTCGTATTCCTAAGGTA
331    V  E  K  A  L  K  D  A  K  M  D  K  S  Q  I  H  D  V  V  L  V  G  G  S  T  R  I  P  K  V
1171  CAATCCCTGCTACAAAACTTCTTCTGTGGTAAGAAACTTAACCTTTCCATCAACCCCGACGAGGCTGTAGCATATGGAGCGGCGGTCCAG
361    Q  S  L  L  Q  N  F  F  C  G  K  K  L  N  L  S  I  N  P  D  E  A  V  A  Y  G  A  A  V  Q
1261  GCAGCTATTTTGAGCGGCGAAACTCACTCTAAGATCCAGGATGTGTTATTGGTAGATGTAGCGCCGCTGTCACTTGGCATAGAAACTGCT
391    A  A  I  L  S  G  E  T  H  S  K  I  Q  D  V  L  L  V  D  V  A  P  L  S  L  G  I  E  T  A
1351  GGAGGAGTCATGACCAAGATAATTGAACGTAACTGCAAAATCCCGTGCAAGCAATCGCAGACATTCACCACCTATTCTGACAACCAGCCT
421    G  G  V  M  T  K  I  I  E  R  N  C  K  I  P  C  K  Q  S  Q  T  F  T  T  Y  S  D  N  Q  P
1441  GCTGTAACTATCCAAGTGTATGAAGGTGAACGAGCAATGACAAAAGATAACAATCTCCTTGGTACTTTCGATCTGACAGGCATACCACCT
451    A  V  T  I  Q  V  Y  E  G  E  R  A  M  T  K  D  N  N  L  L  G  T  F  D  L  T  G  I  P  P
1531  GCACCGCGGGGCGTGCCTAAAATCGACGTCACATTCGACATGGACGCCAACGGTATTCTTAATGTCTCGGCAAAGGAAAACAGTACTGGT
481    A  P  R  G  V  P  K  I  D  V  T  F  D  M  D  A  N  G  I  L  N  V  S  A  K  E  N  S  T  G
1621  CGTAGTAAAAATATCGTGATAAAGAATGATAAGGGACGTTTGTCACAGGCTGAGATTGACAGAATGCTGGCAGAGGCTGAACGATACAAG
511    R  S  K  N  I  V  I  K  N  D  K  G  R  L  S  Q  A  E  I  D  R  M  L  A  E  A  E  R  Y  K
1711  GAAGAGGATGATAAGCAGAGGGAGAGGGTGGCCGCTCGTAACCAACTGGAGACATATGTCTTCAGCGTGCGACAGGCGCTAGATGATGCC
541    E  E  D  D  K  Q  R  E  R  V  A  A  R  N  Q  L  E  T  Y  V  F  S  V  R  Q  A  L  D  D  A
1801  GGAGCGAAGTTACCAGACCAAGACAAAGATTCTGCACGATCACAATGCGACGAAGCGATAAAGTGGTTAGAGAACAACACTTTGGCCGAG
571    G  A  K  L  P  D  Q  D  K  D  S  A  R  S  Q  C  D  E  A  I  K  W  L  E  N  N  T  L  A  E
1891  AAGGAGGAATACGAGCATAAGTTGAAGGAGCTCCAGAGAGTTTGCTCACCCATTATGAGTAAGCTGCATGGTGCTGGTGGTGCCGCCCCT
601    K  E  E  Y  E  H  K  L  K  E  L  Q  R  V  C  S  P  I  M  S  K  L  H  G  A  G  G  A  A  P
1981  GGAGGCCAATCAAATGGACCCACCGTAGAGGAAGTGGATTAAACCTAGTATTAAGAACTAATAGTTATAATTATTTTTAAGACTGATTA
631    G  G  Q  S  N  G  P  T  V  E  E  V  D
2071  ATTTATTTGACTGCAGTGTTAAAACATATGTTTTAATAAACATTATTTAATTTAAAAAAAAAAAAAAAAAAAAAAAAAAAAAA
```

Figure S3. Nucleotide and deduced amino acid sequences of *PmHsp70b*.

```
PmHSC70        : MTTKTPAVGIDLGTTYSCVGVFQYGKVEIIANDQGNRTTPSYVAFTDTERLIGDAAKNQVALN
XP_022130638.1 : MTTKTPAVGIDLGTTYSCVGVFQYGKVDIIANDQGNRTTPSYVAFTDTERLIGDAAKNQVALN
XP_041970467.1 : -MAKAPAVGIDLGTTYSCVGVFQHGKVEIIANDQGNRTTPSYVAFTDTERLIGDAAKNQVAMN
XP_026489199.1 : -MAKAPAVGIDLGTTYSCVGVFQHGKVEIIANDQGNRTTPSYVAFTDTERLIGDAAKNQVAMN
XP_028170304.1 : MATKAPAVGIDLGTTYSCVGVFQHGKVEIIANDQGNRTTPSYVAFTDTERLIGDAAKNQVAMN
                    K PAVGIDLGTTYSCVGVFQ GKVeIIANDQGNRTTPSYVAFTDTERLIGDAAKNQVA6N

PmHSC70        : TAEVYLGKTVQNAVITVPAYFNDSQRQATKDSGTISGLNVLRIINEPTAAAIAYGLDKKGGGE
XP_022130638.1 : TAEVYLGKTVQNAVITVPAYFNDSQRQATKDSGTISGLNVLRIINEPTAAAIAYGLDKKGGGE
XP_041970467.1 : TAEAYLGKTVQNAVITVPAYFNDSQRQATKDSGTISGLNVLRIINEPTAAAIAYGLDKKGGGE
XP_026489199.1 : TAEAYLGKTVQNAVITVPAYFNDSQRQATKDAGTISGLNILRIINEPTAAAIAYGLDKKGTGE
XP_028170304.1 : TAEAYLGKTVQNAVITVPAYFNDSQRQATKDAGTISGLNVLRIINEPTAAAIAYGLDKKGGGE
                 TAE YLGKTVQNAVITVPAYFNDSQRQATKD GTISGLN6LRIINEPTAAAIAYGLDKKGgGE

PmHSC70        : LRRLRTACERAKRTLSSSTQASIEIDSLFEGIDYYTSITRARFEELNADLFRSTMEPVEKSLR
XP_022130638.1 : LRRLRTACERAKRTLSSSTQASIEIDSLFEGIDYYTSITRARFEELNADLFRSTMEPVEKSLR
XP_041970467.1 : LRRLRTACERAKRTLSSSTQASIEIDSLFEGIDFYTSITRARFEELNADLFRSTMEPVEKSLR
XP_026489199.1 : LRRLRTACERAKRTLSSSTQASIEIDSLFEGIDFYTSITRARFEELNADLFRSTMEPVEKSLR
XP_028170304.1 : LRRLRTACERAKRTLSSSTQASIEIDSLYEGIDFYTSITRARFEELNADLFRSTMEPVEKSLR
                 LRRLRTACERAKRTLSSSTQASIEIDSL5EGID5YTSITRARFEELNADLFRSTMEPVEKSLR

PmHSC70        : DLLLLDVTPLSLGIETAGGVMTTLIKRNTTIPTKQTQTFTTYSDNQPGVLIQVFEGERAMTRD
XP_022130638.1 : DLLLLDVTPLSLGIETAGGVMTTLIKRNTTIPTKQTQTFTTYSDNQPGVLIQVFEGERAMTRD
XP_041970467.1 : DLLLLDVTPLSLGIETAGGVMTTLIKRNTTIPTKQTQTFTTYSDNQPGVLIQVFEGERAMTKD
XP_026489199.1 : DLLLLDVTPLSLGIETAGGVMTTLIKRNTTIPTKQTQTFTTYSDNQPGVLIQVFEGERAMTKD
XP_028170304.1 : DLLLLDVTPLSLGIETAGGVMTTLIKRNTTIPTKQTQTFTTYSDNQPGVLIQVFEGERAMTKD
                 DLLLLDVTPLSLGIETAGGVMTTLIKRNTTIPTKQTQTFTTYSDNQPGVLIQVFEGERAMT4D

PmHSC70        : NDAEKYRNEDEKQKETIQAKNALESYCFNMKSTMEDEKLKDKITDSDKQIILDKCNDTIKWLD
XP_022130638.1 : NDAEKYRNEDEKQKETIQAKNALESYCFNMKSTMEDEKLKDKITDSDKQIILDKCNDTIKWLD
XP_041970467.1 : NEAEKYRNEDEKQKETIQAKNSLESYCFNMKSTMEDEKLKDKISDSDKQTILDKCNDTIKWLD
XP_026489199.1 : NEAEKYRNEDEKQKETIQAKNALESYCFNMKSTMEDEKLKDKITDSDKQTILDKCNDTIKWLD
XP_028170304.1 : NEAEKYRNEDDKQKETIQAKNALESYCFNMKSTMEDANLKDKITDADKQTILDKCNDTIKWLD
                 N AEKYRNEDeKQKETIQAKNaLESYCFNMKSTMEDekLKDKI3DsDKQ ILDKCNDTIKWLD

PmHSC70        : EVDD
XP_022130638.1 : EVD-
XP_041970467.1 : EVD-
XP_026489199.1 : EVD-
XP_028170304.1 : EVD-
                 EVD
```

Figure S4. Sequence alignment of PmHSC70 with HSP70 proteins of *Pieris rapae* (XP_022130638.1), *Aricia agestis* (XP_041970467.1), *Vanessa tameamea* (XP_026489199.1) and *Ostrinia furnacalis* (XP_028170304.1).

```
PmHSP70a       : MPAIGIDLGTTYSCVGVWQHGNVEIIANDQGNRTTPSYVAFTDTERLIGDAAKNQVALNPNNT
QWV59544.1     : MPAIGIDLGTTYSCVGVWQHGNVEIIANDQGNRTTPSYVAFTDTERLIGDAAKNQVALNPNNT
XP_038210277.1 : MPAVGIDLGTTYSCVGVWQHGNVEIIANDQGNRTTPSYVAFTDTERLIGDAAKNQVALNPNNT
KAG8115787.1   : MPAIGIDLGTTYSCVGVWQHGNVEIIANDQGNRTTPSYVAFTDTERLIGDAAKNQVALNPNNT
XP_045456590.1 : MPAIGIDLGTTYSCVGVWQHGNVEIIANDQGNRTTPSYVAFTDTERLIGDAAKNQVALNPNNT
                 MPA6GIDLGTTYSCVGVWQHGNVEIIANDQGNRTTPSYVAFTDTERLIGDAAKNQVALNPNNT

PmHSP70a       : YLGSSVRDAVITVPAYFNDSQRQATKDAGAIAGINVLRIINEPTAAALAYGLDKSLKGERNVL
QWV59544.1     : YLGSSVRDAVITVPAYFNDSQRQATKDAGAIAGINVLRIINEPTAAALAYGLDKSLKGERNVL
XP_038210277.1 : YLGSAVKDAVITVPAYFNDSQRQATKDAGAIAGLNVLRIINEPTAAALAYGLDKDLKGERNVL
KAG8115787.1   : YLGTSVRDAVITVPAYFNDSQRQATKDAGAIAGLNVLRIINEPTAAALAYGLDKNLKGERNVL
XP_045456590.1 : YLGTSVRDAVITVPAYFNDSQRQATKDAGAIAGLNVLRIINEPTAAALAYGLDKNLKGERNVL
                 YLG3sV4DAVITVPAYFNDSQRQATKDAGAIAG6NVLRIINEPTAAALAYGLDK LKGERNVL

PmHSP70a       : LRTAAERAKRTLSSSTEATIEIDALYEGMDFYTRVSRARFEELNSDLFRGTLEPVEKALKDAK
QWV59544.1     : LRTAAERAKRTLSSSTEATIEIDALYEGIDYYTRVSRARFEELNSDLFRGTLEPVEKALKDAK
XP_038210277.1 : LRTAAERAKRTLSSSTEATIEIDALYEGIDFYTRVSRARFEELNADLFRGTLEPVEKALKDAK
KAG8115787.1   : LRTAAERAKRTLSSSTEATIEIDALHEGIDFYTRVSRARFEELNADLFRGTLEPVEKALKDAK
XP_045456590.1 : LRTAAERAKRTLSSSTEATIEIDALYEGIDFYTRVSRARFEELNSDLFRGTLEPVEKALKDAK
                 LRTAAERAKRTLSSSTEATIEIDALyEG6D5YTRVSRARFEELN DLFRGTLEPVEKALKDAK

PmHSP70a       : LVDVAPLSLGIETAGGVMTKIIERNAKIPCRQSQTFTTYSDNQPAVTIQVYEGERAMTKDNNL
QWV59544.1     : LVDVAPLSLGIETAGGVMTKIIERNAKIPCRQSQTFTTYSDNQPAVTIQVYEGERAMTKDNNL
XP_038210277.1 : LVDVAPLSLGIETAGGVMTKIIERNAKIPCKQSQTFTTYSDNQPAVTIQVYEGERAMTKDNNL
KAG8115787.1   : LVDVAPLSLGIETAGGVMTKIIERNAKIPCKQSQTFTTYSDNQPAVTIQVYEGERAMTKDNNL
XP_045456590.1 : LVDVAPLSLGIETAGGVMTKIIERNCKIPCKQSQTFTTYSDNQPAVTIQVYEGERAMTKDNNL
                 LVDVAPLSLGIETAGGVMTKIIERNaKIPC4QSQTFTTYSDNQPAVTIQVYEGERAMTKDNNL

PmHSP70a       : ERYKEEDNRQRERVASRNQLESYIFSVKQALDDAGEKLSQEEKNSARNACDEALKWFENNTLA
QWV59544.1     : ERYKEEDNRQRERVATRNQLESYIFSVKQALDDAGEKLGQEDKNTALNACDEALKWFENNTLA
XP_038210277.1 : ERYKEEDSRQRERVAARNQLESYVFSVKQALDEAGEKIGQQDKDTARKACDDALKWLDNNTLA
KAG8115787.1   : ERYKEEDERQRQRVSARNQLESYIFSVKQALDDAGDKLSEQDKQTARNECDEALKWLDNNTLA
XP_045456590.1 : ERYKEEDEKQRQRVAARNQLESYVFSVRQALDDAGSKLSDEDKNTARNVCDEALQWLDNNTLA
                 ERYKEED 4QR2RVa RNQLESY6FSV4QALDdAG K6  2dK 3Arn CDeALkW  NNTLA

PmHSP70a       : PTVEEVDD
QWV59544.1     : PTVEEVD-
XP_038210277.1 : PTVEEVD-
KAG8115787.1   : PTVEEVD-
XP_045456590.1 : PTVEEVD-
                 PTVEEVD
```

Figure S5. Sequence alignment of PmHSP70a with HSP70 proteins of *Pieris rapae* (QWV59544.1), *Zerene cesonia* (XP_038210277.1), *Spodoptera frugiperda* (KAG8115787.1), and *Melitaea cinxia* (XP_045456590.1) .

```
PmHSP70b        : MPAIGIDLGTTYSCVGVWQHGNVEIIANDQGNRTTPSYVAFTDTERLIGDAAKNQVALNPSNT
QWV59542.1      : MPAIGIDLGTTYSCVGVWQHGNVEIIANDQGNRTTPSYVAFTDTERLIGDAAKNQVALNPSNT
XP_045456597.1  : MPAIGIDLGTTYSCVGVWQHGNVEIIANDQGNRTTPSYVAFTDTERLIGDAAKNQVALNPNNT
XP_045456601.1  : MPAIGIDLGTTYSCVGVWQHGNVEIIANDQGNRTTPSYVAFTDTERLIGDAAKNQVALNPNNT
XP_039760955.1  : MPAIGIDLGTTYSCVGVWQHGNVEIIANDQGNRTTPSYVAFTDTERLIGDAAKNQVALNPNNT
                  MPAIGIDLGTTYSCVGVWQHGNVEIIANDQGNRTTPSYVAFTDTERLIGDAAKNQVALNP NT

PmHSP70b        : YLGSSVRDAVVTVPAYFNDSQRQATKDAGVIAGINVLRIINEPTAAALAYGLDKNLKGERNVL
QWV59542.1      : YLGSSVRDAVVTVPAYFNDSQRQATKDAGVIAGINVLRIINEPTAAALAYGLDKNLKGERNVL
XP_045456597.1  : YLGTSVRDAVITVPAYFNDSQRQATKDAGAIAGLNVLRIINEPTAAALAYGLDKNLKGERNVL
XP_045456601.1  : YLGTSVRDAVITVPAYFNDSQRQATKDAGAIAGLNVLRIINEPTAAALAYGLDKNLKGERNVL
XP_039760955.1  : YLGTSVKDAVITVPAYFNDSQRQATKDAGAIAGLNVLRIINEPTAAALAYGLDKSLKGERNVL
                  YLG3SV4DAV6TVPAYFNDSQRQATKDAG IAG6NVLRIINEPTAAALAYGLDKnLKGERNVL

PmHSP70b        : LRTAAERAKRTLSSSTEATVEIDALYEGIDFYTRVSRARFEELNADLFRGTLEPVEKALKDAK
QWV59542.1      : LRTAAERAKRTLSSSTEATVEIDALYEGIDFYTRVSRARFEELNADLFRGTLEPVEKALKDAK
XP_045456597.1  : LRTAAERAKRTLSSSTEATIEIDALYEGIDFYTRVSRARFEELNSDLFRGTLEPVEKALKDAK
XP_045456601.1  : LRTAAERAKRTLSSSTEATIEIDALYEGIDFYTRVSRARFEELNSDLFRGTLEPVEKALKDAK
XP_039760955.1  : LRTAAERAKRTLSSSTEATIEIDALYEGIDFYTRVSRARFEELNSDLFRGTLEPVEKALKDAK
                  LRTAAERAKRTLSSSTEAT6EIDALYEGIDFYTRVSRARFEELN DLFRGTLEPVEKALKDAK

PmHSP70b        : LVDVAPLSLGIETAGGVMTKIIERNCKIPCKQSQTFTTYSDNQPAVTIQVYEGERAMTKDNNL
QWV59542.1      : LVDVAPLSLGIETAGGVMTKIIERNCKIPCKQSQTFTTYSDNQPAVTIQVYEGERAMTKDNNL
XP_045456597.1  : LVDVAPLSLGIETAGGVMTKIIERNCKIPCKQSQTFTTYSDNQPAVTIQVYEGERAMTKDNNL
XP_045456601.1  : LVDVAPLSLGIETAGGVMTKIIERNCKIPCKQSQTFTTYADNQPAVTIQVYEGERAMTKDNNL
XP_039760955.1  : LVDVAPLSLGIETAGGVMTKIIERNSKIPCKQSQTFTTYSDNQPAVTIQVYEGERAMTKDNNL
                  LVDVAPLSLGIETAGGVMTKIIERNcKIPCKQSQTFTTYsDNQPAVTIQVYEGERAMTKDNNL

PmHSP70b        : ERYKEEDDKQRERVAARNQLETYVFSVRQALDDAGAKLPDQDKDSARSQCDEAIKWLENNTLA
QWV59542.1      : ERYKEEDDKQRERVAARNQLETYVFSVRQALDDAGAKLPDQDKDSARSHCDEAIKWLDNNTLA
XP_045456597.1  : ERYKEEDEKQRQRVAARNQLESYVFSVRQALDDAGSKLSDEDKNTARNVCDEALQWLDNNTLA
XP_045456601.1  : ERYKEEDERQQKRVTARNQLESYVFSVRQALDDAGSKLSEEDKNTARNVCDEALQWLDNNTLA
XP_039760955.1  : EKYKEEDEKQRQKVAARNQLESYIFSVRQALDDAGQKLSDADKNSAKSECEEALRWLDNNTLA
                  E4YKEED 4Qr 4VaARNQLE3Y6FSVRQALDDAG KL d DK13A4  CdEA6 WLdNNTLA
```

Figure S6. Sequence alignment of PmHSP70b with HSP70 proteins of *Pieris rapae* (QWV59542.1), *Melitaea cinxias* (XP_045456597.1), *Melitaea cinxia* (XP_045456601.1), and *Pararge aegeria* (XP_039760955.1).
